# Detecting plague-host abundance from space: Using a spectral vegetation index to identify occupancy of great gerbil burrows

**DOI:** 10.1016/j.jag.2017.09.013

**Published:** 2018-02

**Authors:** Liesbeth I. Wilschut, Johan A.P. Heesterbeek, Mike Begon, Steven M. de Jong, Vladimir Ageyev, Anne Laudisoit, Elisabeth A. Addink

**Affiliations:** a Utrecht University, Department of Physical Geography, Heidelberglaan 2, PO Box 80115, 3508 TC Utrecht, The Netherlands; b Utrecht University, Faculty of Veterinary Medicine, Yalelaan 7, 3584 CL Utrecht, The Netherlands; c Ecology Evolution and Genomics of Infectious Disease Research Group, Institute of Integrative Biology, The University of Liverpool, Liverpool, UK; d Anti-Plague Institute, M. Aikimbayev’s Kazakh Science Centre for Quarantine and Zoonotic Diseases, 14 Kapalskaya Street, Almaty 050074, Kazakhstan; e Evolutionary Biology Group, University of Antwerp, 171, Groenenborgerlaan, 2020 Antwerpen, Belgium

**Keywords:** Plague, *Yersinia pestis*, Infectious disease, Great gerbil, Population abundance, NDVI, Object-based image analysis, Segmentation, Random Forest

## Abstract

In Kazakhstan, plague outbreaks occur when its main host, the great gerbil, exceeds an abundance threshold. These live in family groups in burrows, which can be mapped using remote sensing. Occupancy (percentage of burrows occupied) is a good proxy for abundance and hence the possibility of an outbreak. Here we use time series of satellite images to estimate occupancy remotely.

In April and September 2013, 872 burrows were identified in the field as either occupied or empty. For satellite images acquired between April and August, ‘burrow objects’ were identified and matched to the field burrows. The burrow objects were represented by 25 different polygon types, then classified (using a majority vote from 10 Random Forests) as occupied or empty, using Normalized Difference Vegetation Indices (NDVI) calculated for all images. Throughout the season NDVI values were higher for empty than for occupied burrows.

Occupancy status of individual burrows that were continuously occupied or empty, was classified with producer’s and user’s accuracy values of 63 and 64% for the optimum polygon. Occupancy level was predicted very well and differed 2% from the observed occupancy. This establishes firmly the principle that occupancy can be estimated using satellite images with the potential to predict plague outbreaks over extensive areas with much greater ease and accuracy than previously.

## Highlights

• Occupancy of great gerbil burrows can be determined from space using high-resolution satellite images.
• NDVI was successfully used to distinguish between occupied and empty, using the differences in vegetation cover.
• This finding allows predicting plague outbreaks with much greater ease and accuracy, and for much larger areas, than before.

## 1 Introduction

Plague (*Yersinia pestis* infection) is a flea-borne zoonotic disease that is infamous for inducing three pandemics in the past two millennia (Gage & Kosoy, 2005). Currently, the plague agent circulates in rodent populations mainly in Africa, the Americas and Asia, and causes human deaths predominantly in Africa (World Health Organization, 2004). In Kazakhstan, plague occurs, and has been extensively studied, in populations of its main local host, the great gerbil (*Rhombomys opimus*). Great gerbils are burrowing, mainly folivorous rodents that live in semi-desert environments. They live in family groups usually consisting of one male, one or more females and their offspring, and they occupy one burrow per family (Randall et al., 2005).

Presence and absence data of plague from these populations show that plague requires a minimum abundance of great gerbils to be able to spread successfully – the so-called abundance threshold for plague (Davis et al., 2004). The number of gerbils in a burrow varies; a study from 2005, for example, found mean group sizes varied from 3.9 in 1996 to 13.4 in 1998 (Randall et al., 2005). Nonetheless, Davis et al. (2004) found that to monitor fluctuations in great gerbil abundance and predict plague outbreaks accurately, it is not necessary to know the exact number of gerbils in one burrow. Rather, the percentage of the burrows occupied (referred to as the occupancy level) is an effective proxy for abundance and also easier to measure. This is because great gerbils living in the same burrow tend to have the same disease status, i.e. plague is transmitted easily between family members (Davis et al., 2007). Indeed, these occupancy levels can be used to predict plague outbreaks two years in advance (Davis et al., 2004), and they define a ‘percolation threshold’, which, when exceeded, allows plague to spread between occupied burrows across the landscape (Davis et al., 2008, Reijniers et al., 2012).

In the past, and especially since the 1950s, field sampling has been carried out in Kazakhstan to monitor the occupancy level in the field as a means of estimating great gerbil abundance with a view to controlling human plague (Davis et al., 2004). This is expensive in terms of time and manpower, which in turn sets limits on the spatial extent of surveillance. Recently, remote sensing methods were developed that allow highly accurate identification of the presence of burrows (Addink et al., 2010, Wilschut et al., 2013a). However, occupied and unoccupied burrows could not be distinguished with these methods, and occupancy levels (abundance) therefore could not be computed.

The desirability of monitoring plague through tracking gerbil occupancy remotely is thus a focused example of the more general challenge of monitoring animal abundance from space. Although sparse, some examples do exist. Fretwell et al. (2017) estimated the size of an albatross population by counting them manually in 30cm-resolution Worldview-3 imagery. Fretwell et al. (2014) counted whales by supervised and unsupervised classification. And Laliberte and Ripple (2003) counted cattle in an IKONOS image by a computer-aided approach. However, no examples were found on the indirect mapping of a species by detecting traces of an animal like the burrows they built and to determine whether the animals are still present.

Here we take up this more general challenge with the aim ultimately, in this particular case, of making medically relevant predictions of plague risk. Thus, we use high-spatial-resolution remote-sensing images to estimate the gerbils’ occupancy level, aiming to distinguish occupied from empty burrows using variables derived from the images, and subsequently to develop an algorithm to accurately classify a burrows’ occupancy status. To detect a difference in burrow occupancy, we use the behavioural characteristics of the great gerbils. As great gerbils are herbivores, they remove and eat plants and their roots from the surface of their burrow and from its surroundings. This removal of vegetation over the season may be visible in high-spatial-resolution imagery, by using vegetation indices such as the Normalized Difference Vegetation Index (NDVI). As there is a natural change in the NDVI curve of vegetation between spring and autumn, we hypothesize that this curve is different for occupied and empty burrows.

Two objectives are addressed:

1 The first is to assess the NDVI trend of burrow occupancy classes from April to August
2 The second is to use the information obtained to develop an algorithm, using Random Forests, to classify a burrows’ occupancy status, allowing occupancy levels to be estimated from satellite imagery.

## 2 Methods

### 2.1 Field data

**Fig. 1:**
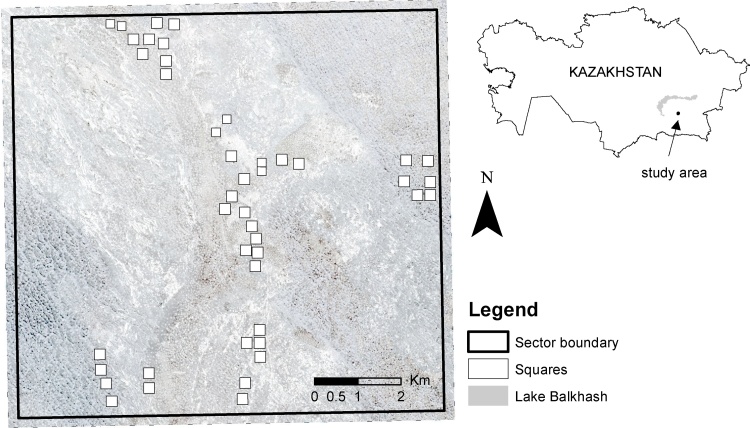
Study area in Eastern Kazakhstan. The 38 field data-collection squares are shown, all located within one sector and projected on WorldView-2 image (DigitalGlobe, 2014).

The study area is located in eastern Kazakhstan, south of Lake Balkhash. Data collection was carried out in 38 squares of 250 by 250 m and six squares of 200 by 200 m in April 2013 and repeated in September 2013. All squares were located in one so-called ‘sector’ (Fig. 1), a monitoring unit of the plague monitoring stations in Kazakhstan, approximately 9.3 km by 9.8 km in size (Wilschut et al., 2013b, Wilschut et al., 2015). The squares are all located in the older part of the river Ili delta consisting of floodplains covered by river sediments, scattered sand dunes and semi-arid vegetation. The diversity of the floodplain was reflected in the square locations. The inaccessibility of the terrain required all squares to be in the vicinity (<1 km) of sand tracks. All burrows inside the squares were mapped by field observation, deriving coordinates from a GPS at the “ecological centre” of the burrow – the location of most intensive visible great gerbil activity. Burrow diameters were measured in two perpendicular axes crossing at the ecological centre (Wilschut et al., 2013a). The occupancy status of each burrow was determined using a standard protocol described in Wilschut et al. (2015) from signs of foraging activity, scent marks, presence of fresh faeces and the recent clearance of burrow entrances (indicated by the presence of freshly turned-over soil). Burrows were classified as either occupied or empty, and all burrows were given a unique code. In addition, the diameter of each burrow was measured.

### 2.2 Satellite data and pre-processing

Five 1.8 m resolution ortho-rectified Worldview-2 images (DigitalGlobe, 2014) with spectral bands Blue, Green, Red and NIR were acquired on April 12th, May 6th, June 5th, July 10th and August 15th 2013. All images were converted to top-of-atmosphere reflectance (Chander et al., 2009). The five images were geo-rectified and subsequently resampled to match the April image using automatic image registration. During evaluation of the data, the July image appeared unsuitable for usage because of many clouds, shade and haze in the image. It was therefore excluded from the analysis. In order to evaluate the overall NDVI trend, the mean NDVI value of the entire sector was calculated for each of the four months.

### 2.3 Evaluation of NDVI values of burrow-occupancy classes

During the two field campaigns, 872 burrows were mapped and categorized in the field as either occupied or empty in April 2013 and in September 2013. This allowed four categories of change between the two months: occupied–occupied (*oo*) in both April and September; empty–empty (*ee*); empty in April-occupied in September (*eo*) and occupied in April-empty in September (*oe*).

In order to assess the NDVI trend of burrow classes, first the area occupied by a burrow was identified by buffering the field coordinates of each burrow centre with the average radius of all burrows. The average burrow diameter mapped in the field in September was 17.8 m. Hence, a circular buffer of 9 m radius was used. The NDVI was calculated for all burrows in all images (April–August 2013). Then, the mean NDVI values for all the burrow-circles were calculated for each month.

### 2.4 Overall burrow identification using remote sensing

To identify the great-gerbil burrows (both occupied and empty) in the sector in the satellite images, the semi-automatic classification method as described in a previous study was used (Wilschut et al., 2013a), with some minor adaptations. Here, this method is only described briefly. First, the field data set was divided into two parts: 50% was used for training the classifier and 50% was used for validation of the burrow classification results. Then, the April 2013 image was segmented into objects using multi-resolution segmentation (Trimble, 2011) at 15 different spatial scales to be able to identify the spatial scale with the highest classification accuracy after classification. Next, the Random Forest classifier (Breiman, 2001) was used to classify the objects in the image as burrow or non-burrow objects. To validate the classification, several indicators were used. First, producer’s, user’s and overall accuracies were calculated (Lillesand et al., 2004). Producer’s accuracy is the percentage of validation burrows that are correctly classified. The user’s accuracy is the percentage of all validation objects classified as burrows that are actually burrows. The overall accuracy is the percentage of classified validation objects (burrows and non-burrow areas) that are classified correctly. Based on the field data, the densities of burrows in the squares were calculated. Then, the overall ratio between the density predicted by the classification and the density observed in the field was calculated to determine the accuracy of the density of the created burrow maps. Finally, the number of objects that intersected burrows multiple times was calculated to determine whether the size of the image objects resembled the size of the field burrows. The optimal segmentation scale was selected by choosing the scale where the combination of the validation variables reached its optimum. The objects that were correctly classified as burrows were processed further.

### 2.5 Occupied burrow classification using remote sensing

**Fig. 2:**
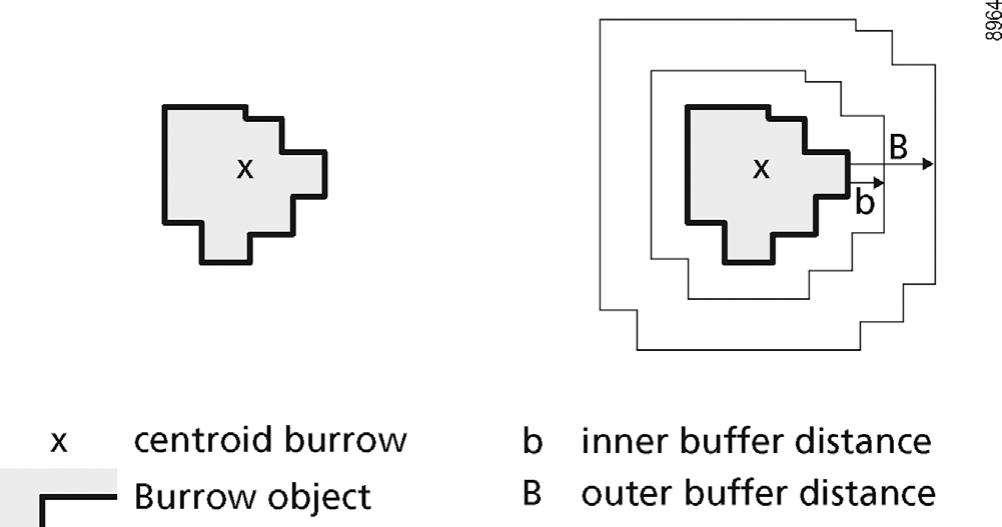
Schematic overview of the sizes of all shapes used for classification of occupied and empty burrows.

The correctly classified burrow objects were divided into the temporal occupancy groups (*oo*, *ee*, *eo* and *oe*) using the field data. The objects that had a constant occupancy status in both April and September (*oo* and *ee*) were used in the Random Forests as these can be expected to have the strongest signal. For each of these objects the NDVI was determined. Furthermore, they were buffered with several distances to determine the NDVI of the surroundings of the burrows, since gerbils forage both on and close to occupied burrows, leading to a different seasonal pattern of vegetation for occupied and empty burrows. Indeed, as the surface of (occupied) burrows is hardly vegetated, the vegetation is visible in the imagery only around the occupied burrows. To enhance the signal from the surrounding vegetation, both the burrow systems combined with their surroundings and the surroundings alone were studied as illustrated in Fig. 2.

The *oo* and *ee* burrow objects were buffered with distances of 5, 7.5, 10, 12.5, 15, 20, 25 and 30 m, to create polygons of different sizes. For all these polygons, mean NDVI values were determined for the four images. Next, donuts were created to represent the surroundings of burrows (Table 1, Fig. 2), and NDVIs were calculated for these donut polygons. The outer buffer distance as well as the inner buffer distance of the donut could have different diameters. For example, the donuts p^15-5^ were created by symmetrical differencing (subtracting the polygons) buffered with a distance of 5 m from polygons buffered with a distance of 15 m. Apart from the mean NDVI values determined for every image, several derivative, temporal variables were also calculated for both the polygons and the donuts of the various sizes. These variables include the absolute temporal differences in NDVI values, such as |(NDVI August minus NDVI July)| and the normalized temporal differences in NDVI variables, such as NDVI August minus NDVI July divided by|(NDVI August plus NDVI April)|. For a full list of the variables used see Table 1, Table 2.

**Table 1:** Overview of the shapes used for classification. See also Fig. 2.

Spatial shape types tested
Polygons (p) with buffer B (radius m) [P^B^]
p^0^ (no buffer), p^5^, p^7.5^,p^10^,p^12.5^, p^15^, p^20^, p^25^ and p^30^
Donuts [p^B-b^]
p^10-0^; p^15-0^; p^20-0^; p^25-0^;mp^30-0^;
p^15-5^; p^20-5^; p^25-5^; p^30-5^; p^15-7.5^; p^20-7.5^; p^25-7.5^; p^30-7.5^
p^20-10^; p^25-10^; p^30-10^

**Table 2:** Overview of the NDVI variables used for classification.

Mean	Absolute difference	Normalized difference	Mean monthly change
April			
May			
June			
August			
	April–May	April–May	
	April–June	April–June	
	April–August	April–August	
	May–June	May–June	
	May–August	May–August	
	June–August	June–August	
			May–August
			June–August

For the classification, the *ee* and *oo* object groups were split into a training set and validation set, by taking a random sample. The training set contained equal numbers of *oo* and *ee* objects (both 77 objects) to have equal a-priori probabilities for both groups. The validation set contained 77 *oo* and 329 *ee* objects. Classification was carried out using Random Forests. A Random Forest is a robust statistical classifier that makes a prediction of a test set based on observations from a training set, using multiple decision trees (Breiman, 2001). For further details, see (Diaz-Uriarte, 2007, Cutler et al., 2007, Verikas et al., 2011, Rodriguez-Galiano et al., 2012). The Random Forests built in this study used 10,000 trees, which is a sufficient number to ensure the Out-of-Bag (OOB) mean prediction error does not increase or decrease anymore if the number of trees is increased (Breiman, 2001). Each Random Forest was optimized by removing the least predictive variables using the *VarSelRF* R package (Diaz-Uriarte, 2007). VarselRF uses backward elimination. The function keeps dropping variables until the OOB error becomes larger than the initial OOB error. In this way the number of variables is reduced, but not at the cost of the OOB error. For other settings the default values were used (Liaw and Wiener, 2002). For each polygon or donut-like-polygon set, 25 sets in total (nine sizes of full polygons and 16 sizes of donuts), ten Random Forests were built to avoid overfitting on a randomly selected training set.

To determine if the full polygons and the donuts performed differently a Student *t*-test was applied. For both types the mean and standard deviation of the overall classification accuracy was determined based on each size and each Random Forest (i.e. 9*10 full polygon accuracy values and 16*10 donut accuracy values). These values were then fed into the Student *t*-test.

### 2.6 Occupancy estimation

To be able to predict plague, it is essential to have good predictions of the occupancy level within a region. The percentage of occupied burrows is thus an important variable to derive from the burrow occupancy classification. We used the ratio between the observed occupancy and the predicted occupancy as an accuracy indicator of the estimation. For the optimal polygon set, this ratio was calculated for each Random Forest.

### 2.7 Validation

To ensure reliable classification accuracies, each of the polygon sets was classified ten times, each time using randomly drawn 50% training and validation samples. Mean producer’s, user’s and overall accuracies and their standard deviations were calculated from these ten runs. Both the producer’s accuracies and user’s accuracies for occupied and empty burrows were calculated. Current plague prediction models use the percentage of occupied burrows for a region and the ultimate objective is therefore an accurate prediction of regional occupancy. The ratio between predicted and observed occupancy was calculated to validate this (values closest to one being the best).

## 3 Results

### 3.1 NDVI values of burrow classes using field locations

**Fig. 3:**
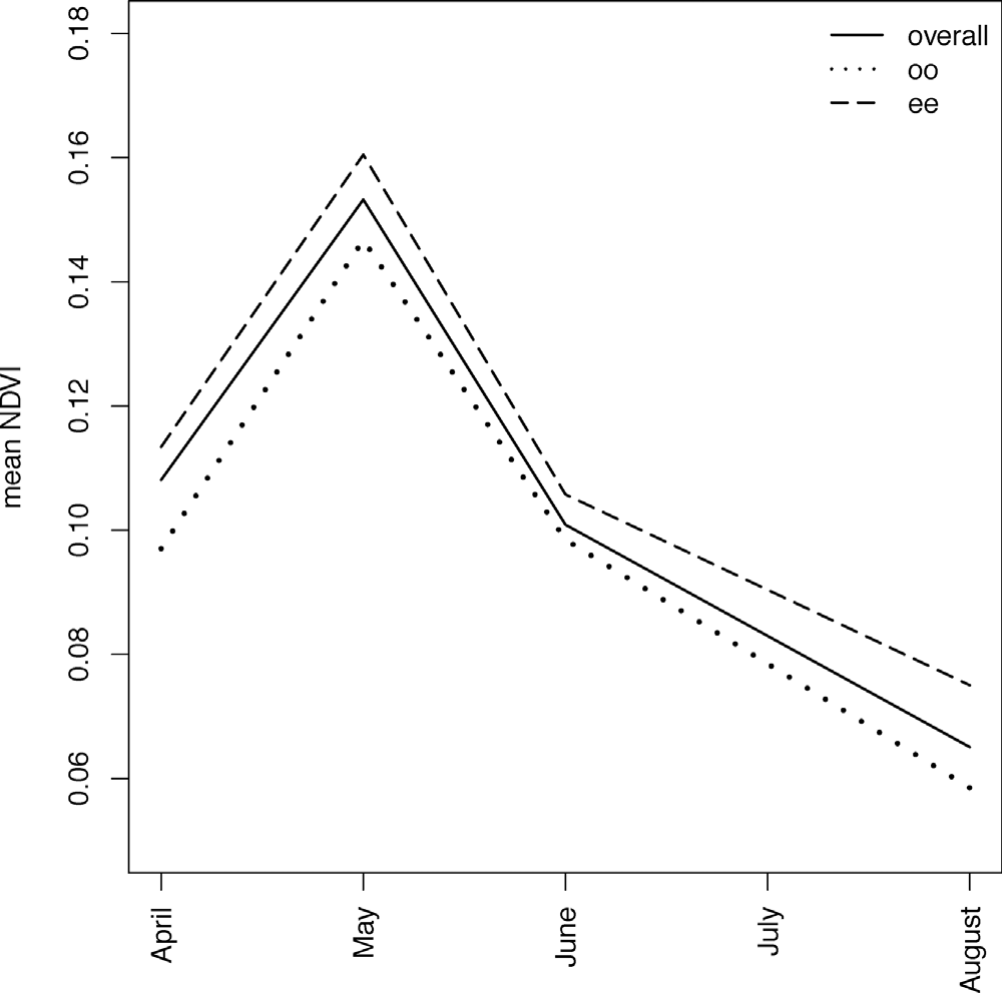
NDVI trends calculated for the entire sector using all pixels (n = 27,783,504), the continuously occupied burrows (*oo*; number of pixels = 13038) and the continuously empty burrows (*ee*; number of pixels = 31887).

In total, 872 burrows were mapped in both April and September 2013, of which 166 were continuously occupied (*oo*), 406 continuously empty (*ee*), 31 occupied-empty (*oe*) and 269 empty-occupied (*eo*). The mean NDVI values for the images, calculated using all pixels in the sector (number of pixels = 27,783,504) (Fig. 3), showed that NDVI reached a peak in May (NDVI = 0.15) and then gradually decreased. Mean NDVI values for the continuously occupied (*oo*; number of pixels = 13038) and continuously empty (*ee*; number of pixels = 31887) burrows (based on 9 m radius of the circles) showed a similar pattern from April to June, although the *ee* burrows had higher and the *oo* burrows lower mean values. Between June and August there was a slight further deviation from the overall trend: the *ee* NDVI values decreased less rapidly.

**Fig. 4:**
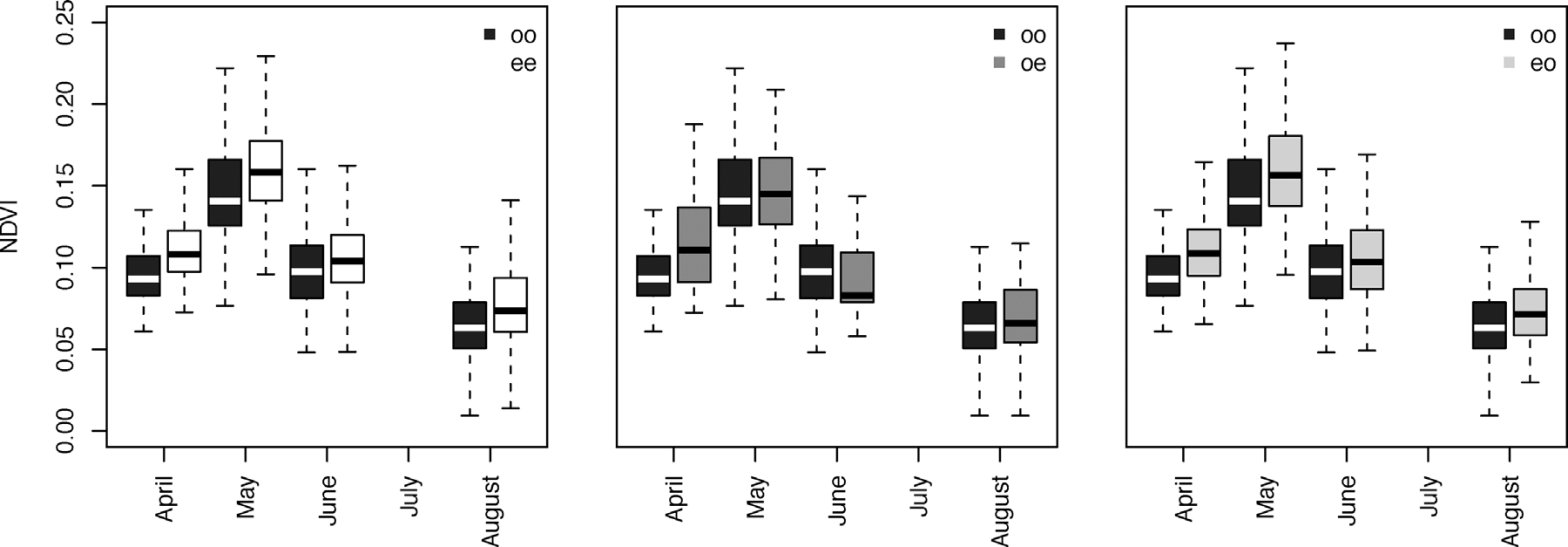
Left: Box plots of NDVI values for continuously occupied *(oo)* burrows and continuously empty *(ee)* burrows. *Middle*: Similar, but then for *oo* and occupied-empty (*oe)* burrows. Right: Similar, but then for *oo* and empty-occupied (*eo)* burrows.

In all months the *oo* burrows had lower values of mean NDVI than the *ee* burrows (Fig. 3, Fig. 4). The occupied-empty class followed a less constant pattern (Fig. 4), possibly because this was the least numerous category (31 burrows). The *eo* class on the other hand followed a similar pattern as the *ee* class and had larger NDVI values than the *oo* class in all months.

### 3.2 Overall burrow identification using remote sensing

The burrow classification resulted in producer’s, user’s and overall accuracies of 91%, 82% and 90%, respectively. The accuracy of predicted density, i.e. the ratio between the density predicted by the classification and the density observed in the field, was 1.49.

### 3.3 Classification of burrow occupancy

The highest classification accuracies were obtained when using full polygons without a buffer (p^0^, see Table S.1 in the Supplementary material). Of the ten Random Forests built for this polygon-set, the best results were obtained using the variables NDVI April, NDVI May and NDVI August. This resulted in producer’s and user’s accuracies of occupied burrows of 64.3% and 63.3%, respectively. The producer’s and user’s accuracies for empty burrows were 62.7% and 64.0%. Overall accuracy of the classification was 63.5%. All classification accuracies for the ten Random Forests are given in Table S.2 in the Supplementary material. The standard deviation of the overall classification accuracy of the ten runs is 3.8%, which shows that the method is robust, even when slightly different variables are used.

In total, eight different NDVI variables were selected by the Random Forests in the ten runs. Most often selected were: NDVI April (9 out of 10 runs), NDVI August (8 out of 10), NDVI May (3 out of 10), (NDVI May–NDVI August)_normalized_ (3 out of 10) and (NDVI June–NDVI August)_normalized_ (3 out of 10).

No significant differences were found between the classification accuracies of objects and donuts (Student *t*-test, p = 0.81). The accuracies of the full polygons with buffer and the accuracies of the donuts are shown in Table S.1.

**Fig. 5:**
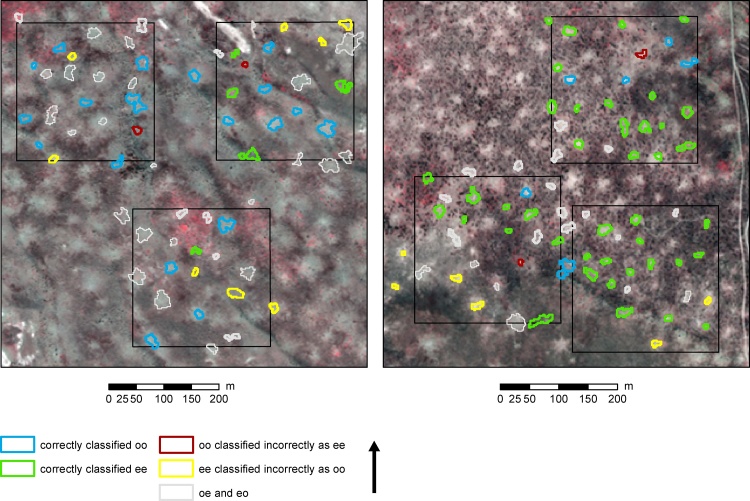
Worldview image showing identified burrows and their occupancy. Polygons, regardless their colour, show burrow locations. The occupancy of the burrows is indicated with colours. The left image shows a dune-rich area; the right image shows an area with fluvial sediments.

As an indication of the spatial variation in the results of the classification, the classification results are shown for two areas in Fig. 5. In a more dune-rich area (Fig. 5 – left), where occupancy is relatively high, the identification of *oo* burrows was successful: only two *oo* burrows were misclassified (shown in red in Fig. 5). On the other hand, nine *ee* burrows were incorrectly classified as *oo* (yellow in Fig. 5). In a floodplain area (Fig. 5 – right) the occupancy is much lower and it can be seen that the *ee* burrows are almost all correctly classified (except for six). Unfortunately, the set-up of our study does not support an extensive characterization of the spatial variation.

### 3.4 Regional occupancy

The regional occupancy level is an essential variable to predict plague outbreaks. The full results for the ratios of predicted occupancy to observed occupancy are shown in Tables S.1 and S.2. Summarizing, the ratios calculated based on only validation data show that occupancy level can be predicted very well: a mean ratio of 1.02 for the optimum polygon with a range among the 10 Random Forests from 0.82 to 1.17.

## 4 Discussion and conclusions

A gerbil-burrow-occupancy threshold (i.e. a percentage of burrows occupied) exists for plague to spread and persist across large regions of Central Asia (Davis et al., 2008). Monitoring great gerbil abundance has the potential to determine when this threshold comes close to being exceeded, and hence predicting when the risk is increasing of plague outbreaks in the great gerbil and ultimately in the human population. Here, we have established that there are differences in NDVI values between occupied and empty great gerbil burrows, and therefore that satellite-image-based prediction of occupancy and abundance are possible. We note, particularly, that while the ability of our methods to distinguish between occupied and empty individual burrows is important, the ultimate aim would be to derive useful estimates of the level of burrow occupancy, as a proxy for gerbil abundance, allowing prediction of plague outbreaks.

Estimating animal abundance is important in many areas of ecology, including disease ecology, where the abundance of animal hosts influences the dynamics of infectious agents (Keeling, 1999, Keeling and Gilligan, 2000). Animal abundance has been estimated using a range of field-based methods (Schwarz and Seber, 1999). Remote-sensing-based methods based on proxies or surrogates have also been developed, but observed patterns cannot usually be ascribed to one species alone (Leyequien et al., 2007), relying, for example, on habitat suitability mapping (Prins et al., 2005) or assessing species richness through spatial heterogeneity assessment (Seto et al., 2004). In particular, if the purpose is to predict infectious disease outbreaks, the spatial resolution of these remote-sensing studies is likely to be insufficient because no individual animals or subpopulations are mapped, and only rather crude estimates are given. In this study, by contrast, we have presented a remotely-sensed, proxy method to estimate animal abundance with an arguably useful level of accuracy and with high spatial detail. This is in contrast to other studies that, for example, found relationships between abundance and NDVI (Zinner et al., 2001), but failed to predict animal distribution. In studies where the abundance of vector species was predicted, the spatial unit is usually much larger than the animal observed (Tran et al., 2008).

Burrow-NDVI values deviated from the sector-mean NDVI values, indicating that the great gerbils locally have a profound effect on the vegetation dynamics. On *ee* burrows (unoccupied throughout the study period), the NDVI values were higher than the sector-mean NDVI. This may be due to past occupation by gerbils making the soil more fertile, as it appears to have done for burrowing sand rats (*Psammomys obesus)* in Egypt (El-Bana, 2009) and great gerbils in China (Xu et al., 2012). Further, on great-gerbil burrows, after one year without great gerbils, vegetation cover increased from 10 to 15% to 30–40% (Naumov and Lobachev, 1975). The lower NDVI values of the *oo* burrows from April to June are likely to have arisen because the great gerbils remove plants and shrubs for consumption or storage. Similarly, between June and August, the NDVI of the *oo* class decreased faster than that of the *ee* class.

The distinction between occupied and empty burrows worked best using objects without a buffer. Although gerbils do forage in the surroundings of their burrow, this is apparently not sufficient to be reflected in NDVI values. Alternatively, it may be that the NDVI difference is much stronger on the burrows themselves and including the surroundings in the analysis weakens the NDVI signal.

Several NDVI variables were included in the Random Forests. The analysis showed that the combination of NDVI April, NDVI May and NDVI August resulted in the highest accuracies. In the ten runs, NDVI April, NDVI May and NDVI August were also the three most often selected variables. These three variables have the most differentiating power and are hence recommended in future analyses. This makes the method developed here modest with respect to investments: effectively only satellite images of three months are necessary, and field work in some strategic locations which represent landscape variability, suffices. Notably, *change rates* of NDVI values over time did not prove necessary for distinguishing occupied from empty burrows.

The approach presented here first identified burrows and then further discriminated between occupied and empty burrows. Both steps are subject to uncertainties. When identifying burrows irrespective of occupancy, false-positive (objects wrongly classified as burrows) as well as false-negative (missed burrow) errors occur. The burrows that are most likely to result in a false-negative error are the empty burrows, because they show less contrast with the surroundings (Wilschut et al., 2013a). This may lead to an overestimation of occupancy. The classification into occupied and empty burrows was made difficult, in part, because although the mean NDVI values of occupied and empty burrows differed, confidence intervals showed substantial overlap. Successful mapping of burrow occupancy may also have been hampered by the low occupancies in 2013. Mean occupancy as estimated in the field was 22.8% in April and 48.7% in September. Low occupancies likely indicate that the number of great gerbils per burrow is lower than the long-term average, leading in turn to less grazing pressure than at higher occupancies.

The producer’s and user’s accuracies for occupied (64% and 63%, respectively) and empty burrows (63% and 64%, respectively) were not as high as for burrow locations irrespective of occupancy (91% and 82%, respectively), but were nonetheless encouraging given the increased difficulty of the task. However, if the focus is shifted from individual burrows to occupancy levels, which are important because they relate directly to the spread of plague (Davis et al., 2004), then the average ratio between the predicted and observed occupancy levels (1.02) is close to unity (perfect prediction).

In general, it is likely that when occupancies are stable for a number of years, they can be better predicted, because the *oe* and *eo* classes will be small, and therefore the differences between occupied and empty burrows will be more pronounced. The years when it is more difficult to predict occupancy with high accuracy are those when the overall population of great gerbils is expanding (as was the case here) or contracting. Nonetheless, even in such cases, our estimates are valuable since in practice, previous studies of abundance thresholds for plague spread (Davis et al., 2004, Davis et al., 2007, Davis et al., 2008, Reijniers et al., 2012) have used annual estimates of burrow occupancy, combining spring and autumn values. Given the potentially enormous benefits of remote, as opposed to direct, estimation of occupancy, in terms of manpower, accessibility of sites and the extent of the area covered, the ratio of 1.02 that we found is a very encouraging level of accuracy.

In practical terms, based on our results, we recommend the following method for mapping occupancy in future studies. Satellite images should be acquired in April, May and August. Whether the same Random Forest with the training data from 2013 can be used in subsequent years depends on the NDVI values. From year to year the NDVI can vary due to variations in for example precipitation. To account for this, the sector-mean NDVI values should first be compared with the sector-mean NDVI values obtained from the new satellite images, preferably both in April and in August. If the new NDVI values resemble the NDVI of the 2013 images, the Random Forest used for the 2013 data can in theory be used to predict occupancy. If not, for example a transformation could be applied to the NDVI in the new images to account for this.

To conclude, occupied and empty burrows have different NDVI signatures and this characteristic has been used to classify occupied burrows, which are a proxy for gerbil abundance, and from which the occupancy level can be derived. This study was possible because, although the great gerbils make daily foraging movements, they have a stable residence, which can be monitored from space. Classifying occupied burrows can most likely also be applied to the estimation of abundance of other burrowing rodents in environments with short vegetation. More importantly, the occupancy level of burrows is an important predictor of plague outbreaks and the spatial distribution of occupied burrows can be used to more accurately model the spatial dynamics of plague. This is an encouraging step towards a monitoring approach for the future that can predict plague outbreaks because fluctuations in abundance of the wildlife host can be monitored remotely over extensive areas.
