# Supplementary material for: Detecting plague-host abundance from space: Using a spectral vegetation index to identify occupancy of great gerbil burrows
Source: Int J Appl Earth Obs Geoinf. 2018 Feb;64:249–55. doi: 10.1016/j.jag.2017.09.013 (PMC5763245; doi:10.1016/j.jag.2017.09.013)
Supplement: Supplementary file 1 [file mmc1.docx]

**Supplementary material to “Detecting plague-host abundance from space: using a spectral vegetation index to identify occupancy of great gerbil burrows”**

**Table S.1 Classification accuracies of polygon-objects, both regular and donut-shaped, with varying buffer lengths. For every polygon-object-set, for example p^5^, ten Random Forests were constructed. The mean accuracies (producer’s and user’s accuracy of both occupied and empty burrows, and the overall accuracy) of these ten Random Forests are given with their standard deviations. The ratio between the predicted occupancy and the observed occupancy is also shown. Polygon set p^0^ (shaded) shows the highest overall mean accuracy. The performance of the individual forests for this set are shown in Table S.2.**

| **Polygon**  **set** | **Mean**  **producer’s**  **accuracy (PA) *oo***  **burrows** | | **Mean**  **user’s**  **accuracy (UA)**  ***oo* burrows** | | **Mean producer’s**  **Accuracy (PA) *ee***  **burrows** | | **Mean**  **user’s**  **accuracy**  **(UA) *ee***  **burrows** | | **Mean**  **overall**  **accuracy (OA)** | | **Mean of**  **ratios**  **predicted occupancy**  **/**  **observed occupancy**  **(using validation data only)** |
| --- | --- | --- | --- | --- | --- | --- | --- | --- | --- | --- | --- |
| **Regular polygons** | **PA (%)** | **± SD**  **(%)** | **UA (%)** | **± SD**  **(%)** | **PA (%)** | **± SD**  **(%)** | **UA (%)** | **± SD**  **(%)** | **OA (%)** | **± SD (%)** |  |
| P^0^ | 64.3 | 7.4 | 63.3 | 3.8 | 62.7 | 7.4 | 64.0 | 3.8 | 63.5 | 3.9 | 1.02 |
| P^5^ | 46.4 | 4.4 | 48.0 | 2.3 | 49.9 | 4.4 | 48.2 | 2.3 | 48.1 | 2.1 | 0.96 |
| p^7.5^ | 54.9 | 6.1 | 57.8 | 6.8 | 59.2 | 6.1 | 56.6 | 6.8 | 57.1 | 6.2 | 0.96 |
| p^10^ | 61.9 | 5.4 | 60.7 | 4.5 | 59.5 | 5.4 | 61.0 | 4.5 | 60.7 | 3.8 | 1.02 |
| p^12.5^ | 59.2 | 5.3 | 57.7 | 3.6 | 56.5 | 5.3 | 58.1 | 3.6 | 57.9 | 3.5 | 1.03 |
| p^15^ | 57.9 | 4.6 | 56.8 | 3.0 | 55.7 | 4.6 | 56.9 | 3.0 | 56.8 | 2.8 | 1.02 |
| p^20^ | 51.4 | 7.6 | 51.7 | 4.6 | 51.9 | 7.6 | 51.7 | 4.6 | 51.7 | 4.7 | 0.99 |
| p^25^ | 57.0 | 6.3 | 55.6 | 3.4 | 54.3 | 6.3 | 55.9 | 3.4 | 55.6 | 3.6 | 1.03 |
| p^30^ | 56.0 | 6.8 | 58.5 | 4.1 | 60.1 | 6.8 | 57.8 | 4.1 | 58.1 | 3.5 | 0.96 |
| **Donut-shaped polygons** |  |  |  |  |  |  |  |  |  |  |  |
| p^10-0^ | 62.3 | 4.8 | 61.5 | 4.3 | 60.6 | 4.8 | 61.7 | 4.3 | 61.5 | 3.7 | 1.02 |
| p^15-0^ | 56.2 | 4.9 | 55.5 | 2.4 | 54.7 | 4.9 | 55.5 | 2.4 | 55.5 | 2.3 | 1.02 |
| p^20-0^ | 51.7 | 8.8 | 53.9 | 4.9 | 55.8 | 8.8 | 53.7 | 4.9 | 53.8 | 4.8 | 0.96 |
| p^25-0^ | 54.7 | 7.5 | 55.3 | 3.7 | 55.8 | 7.5 | 55.3 | 3.7 | 55.3 | 3.7 | 0.99 |
| p^30-0^ | 54.4 | 8.4 | 57.3 | 4.2 | 59.2 | 8.4 | 56.7 | 4.2 | 56.8 | 3.8 | 0.95 |
| p^15-5^ | 58.7 | 5.8 | 56.6 | 3.3 | 54.8 | 5.8 | 57.1 | 3.3 | 56.8 | 3.4 | 1.04 |
| p^20-5^ | 50.9 | 8.5 | 52.6 | 4.2 | 54.2 | 8.5 | 52.5 | 4.2 | 52.5 | 4.2 | 0.97 |
| p^25-5^ | 57.3 | 6.6 | 56.5 | 4.0 | 55.8 | 6.6 | 56.8 | 4.0 | 56.6 | 4.1 | 1.01 |
| p^30-5^; | 56.6 | 8.4 | 57.2 | 4.8 | 57.3 | 8.4 | 57.0 | 4.8 | 56.9 | 4.2 | 0.99 |
| p^15-7.5^ | 60.5 | 7.6 | 57.3 | 3.5 | 54.9 | 7.6 | 58.4 | 3.5 | 57.7 | 3.7 | 1.06 |
| p^20-7.5^ | 50.9 | 7.1 | 53.3 | 3.3 | 55.5 | 7.1 | 53.1 | 3.3 | 53.2 | 3.3 | 0.95 |
| p^25-7.5^ | 55.1 | 4.0 | 54.5 | 3.2 | 53.6 | 4.0 | 54.3 | 3.2 | 54.4 | 2.9 | 1.01 |
| p^30-7.5^ | 56.9 | 7.8 | 57.0 | 4.2 | 57.0 | 7.8 | 57.1 | 4.2 | 56.9 | 3.9 | 1.00 |
| p^20-10^ | 49.7 | 7.7 | 52.5 | 4.1 | 55.1 | 7.7 | 52.3 | 4.1 | 52.4 | 4.1 | 0.95 |
| p^25-10^ | 55.3 | 6.4 | 55.6 | 3.6 | 55.7 | 6.4 | 55.6 | 3.6 | 55.5 | 3.6 | 1.00 |
| p^30-10^ | 57.4 | 8.8 | 58.1 | 5.5 | 58.4 | 8.8 | 58.0 | 5.5 | 57.9 | 5.2 | 0.99 |

**Table S.2 Classification accuracies of polygon-objects p^0^ (polygon objects with no buffer) for the ten Random Forests. Producer’s and user’s accuracy of both occupied and empty burrows, and the overall accuracy are shown. The ratio between the predicted occupancy and the observed occupancy is also shown.**

| **Random**  **Forest**  **number** | **Producer’s**  **accuracy**  ***oo***  **burrows**  **(%)** | **User’s**  **accuracy**  ***oo***  **burrows**  **(%)** | **Producer’s**  **accuracy**  ***ee***  **burrows**  **(%)** | **User’s**  **accuracy**  ***ee***  **burrows**  **(%)** | **Overall**  **accuracy**  **(%)** | **predicted occupancy/**  **observed occupancy**  **(using validation data only)** |
| --- | --- | --- | --- | --- | --- | --- |
| 1 | 55.8 | 58.1 | 59.7 | 57.5 | 57.8 | 0.96 |
| 2 | 74.0 | 71.3 | 70.1 | 73.0 | 72.1 | 1.04 |
| 3 | 72.7 | 62.2 | 55.8 | 67.2 | 64.3 | 1.17 |
| 4 | 66.2 | 62.2 | 59.7 | 63.9 | 63.0 | 1.06 |
| 5 | 50.6 | 61.9 | 68.8 | 58.2 | 59.7 | 0.82 |
| 6 | 62.3 | 61.5 | 61.0 | 61.8 | 61.7 | 1.01 |
| 7 | 71.4 | 61.1 | 54.5 | 65.6 | 63.0 | 1.17 |
| 8 | 63.6 | 64.5 | 64.9 | 64.1 | 64.3 | 0.99 |
| 9 | 62.3 | 62.3 | 62.3 | 62.3 | 62.3 | 1.00 |
| 10 | 63.6 | 68.1 | 70.1 | 65.9 | 66.9 | 0.94 |
| **Average**  **(±SD)** | **64.3**  **± 7.4** | **63.3**  **± 3.8** | **62.7**  **± 7.4** | **64.0**  **± 3.8** | **63.5**  **± 3.9** | **1.02**  **± 0.10** |
